# Assessment of fear and anxiety associated behaviors, physiology and neural circuits in rats with reduced serotonin transporter (SERT) levels

**DOI:** 10.1038/s41398-019-0368-y

**Published:** 2019-01-22

**Authors:** Philip L. Johnson, Andrei I. Molosh, Lauren M. Federici, Cristian Bernabe, David Haggerty, Stephanie D. Fitz, Eugene Nalivaiko, William Truitt, Anantha Shekhar

**Affiliations:** 1 Department of Anatomy and Cell Biology, Indiana University School of Medicine, Indianapolis, IN USA; 2 Stark Neurosciences Research Institute, Indiana University School of Medicine, Indianapolis, IN USA; 3 Department of Psychiatry, Indiana University School of Medicine, Indianapolis, IN USA; 4 Program in Medical Neurosciences, Indiana University School of Medicine, Indianapolis, IN USA; 5 School of Biomedical Sciences and Pharmacy, University of Newcastle, Newcastle, NSW Australia

## Abstract

Genetic variation in serotonin transporter (SERT) that reduces transcriptional efficiency is associated with higher anxiety and fear traits and a greater incidence of post traumatic stress disorder (PTSD). Although previous studies have shown that rats with no expression of SERT (SERT^−/−^) have increased baseline anxiety behaviors, SERT^+/−^ rats with low SERT expression (and more relevant to the clinical condition with low SERT expression) do not. Yet, no systematic studies of fear acquisition/extinction or their underlying neural mechanisms have been conducted in this preclinical genetic SERT^+/−^ model. Here we sought to determine if SERT^+/−^ or SERT^−/−^, compared to wildtype, rats would show exacerbated panic responses and/or persistent conditioned fear responses that may be associated with PTSD or phobia vulnerability. Results: Only SERT^−/−^ rats showed increased baseline anxiety-like behaviors with heightened panic respiratory responses. However SERT^+/−^ (also SERT^-/-^) rats showed enhanced acquisition of fear and delayed extinction of fear that was associated with changes in serotonergic-related genes (e.g., reduced 5-HT1A receptor) and disrupted inhibition within the basolateral amygdala (BLA). Furthermore, the disrupted fear responses in SERT^+/−^ rats were normalized with 5HT1A antagonist infusions into the BLA. Enhanced acquisition and failure to extinguish fear memories displayed by both SERT^−/−^ and SERT^+/−^ rats are cardinal symptoms of disabling anxiety disorders such as phobias and PTSD. The data here support the hypothesis that reduced SERT function is a genetic risk that disrupts select gene expression and network properties in the amygdala that could result in vulnerability to these syndromes.

## Introduction

Post traumatic stress disorder (PTSD) and panic disorder (PD) represent some of the most severe and disabling trauma/anxiety related disorders^1^. Severe and or repeated psychological trauma can result in PTSD, which is associated with symptoms such as persistent fear associated memories (e.g., flashbacks) that can trigger panic attacks (PA)^2^ and lead to avoidance of activities, places and stimuli that produce these flashbacks. In regards to PD, the cardinal symptoms are unexpected and recurrent (uPA)’s that occur in the absence of a clear external trigger and are estimated to account for ~40% of PA’s^3^. Recurrent unexpected PAs can produce a conditioned avoidance response that occurs when people with PD are traumatized by the uPA’s and begin to fear situations that are associated with the uPA’s. This can then induce expected PA’s^3^ and agoraphobia in ~50% of humans with PD^4^. Although unexpected, PA’s can be reliably induced in humans with PD with viscerosensory associated stimuli such as 5–7% hypercapnic gas exposure^5–7^, which do not induce PAs in healthy controls at these concentrations. The relevance is that subtle increases in CO_2_ in the blood caused by hypoventilation or holding one’s breath result in acidosis in peripheral and central brain structures that initially results in an increase in respiration activity to help “blow off” excess CO_2_ [see review^8^]. However, a sense of suffocation occurs if CO_2_ levels continue to increase, which also produces adaptive behavioral and autonomic responses that recapitulate symptoms of PA’s. For example, higher concentrations of 20% CO_2_ will induce symptoms consistent with PA’s in healthy humans^9^ and also induce concentration dependent increases in fear in healthy humans that is greater (more severe) in humans with PD^10^. Thus there is evidence that the initial PD pathology is associated with an alteration in central neural pathways which renders them susceptible to uPA’s when exposed to otherwise non panic inducing viscerosensory stimuli^11^.

In the US, there is evidence that the risk of being exposed to a severe trauma could be as high as 75%^12^, yet PTSD occurs in only about 7% of the population^13^ [see also review^14^]. Therefore the risk of being traumatized is much higher than the prevalence of PTSD. This suggests that the majority of people have some resiliency to traumatic events, but others may be more vulnerable (e.g., genetic contributions). In regards to PD, the prevalence in the general population is ~2–5%^4,15^ with a strong heritability in first degree relatives (~11%) and monozygotic twins (30–40%)^16,17^, suggesting that in this case there are clear genetic contributions. Consistent with this hypothesis is that in humans with PD a meta-analysis of candidate genes identified several replicable candidate genes^18^ such as serotonin transporter (SERT) polymorphisms which is associated with severity of PAs^18,19^. SERT reuptake inhibitors (SSRIs) are also the gold standard for treating severe anxiety/trauma disorder such as PD and PTSD^20,21^. In 1996 Lesch and colleagues assessed anxiety traits in humans carrying a specific polymorphism in the promoter region of the SERT gene, where the dominant short (s) allelic variant reduces transcriptional efficiency of the SERT^22^, which is evidenced by reduced brain SERT mRNA levels^23^, and reduced binding^24^. It has been determined that humans with this SERT polymorphism have increased anxiety-associated traits^22^ and enhanced fear conditioned responses^25,26^. This specific SERT ss-allele polymorphism is also associated with a higher risk of PTSD in humans exposed to high trauma^27^, and although it is not associated with a higher risk for PD^27^ it is associated with more severe PA’s^29^.

Among anxiety circuits, the basolateral amygdala (BLA) is a critical site where serotonin plays an important role in modulating anxiety and fear responses and is highly responsive to stressful stimuli^30–34^. The BLA also plays a critical role in fear conditioned responses [see reviews^35,36^]. Humans with the SERT polymorphism have enhanced baseline amygdala activity and reactivity to fearful stimuli that is likely a contributing factor to increased anxiety and fear-associated traits^37,38^. However humans with atrophy of the amygdala region, whom have disrupted fear conditioned responses, display similar if not worse panic responses to a 20%CO_2_ challenge^39^, and in one case study an intact amygdala was not necessary for a subject to experience PA’s^40^. Consistent with this finding, Schruers and colleagues determined that humans with the SERT s-allele polymorphism displayed less fear to a panicogenic CO_2_ challenge^41^. Taken together this suggests that the SERT polymorphism may not be associated with initial unexpected PAs that are the initial phase of PD, but may predispose them to the later development of amygdala regulated conditioned responses such as agoraphobia.

Similar to human SERT polymorphism, a heterozygous null mutation of the SERT gene (SERT^+/−^) is also associated with reduced transcriptional efficiency in rats^42^. Rats with complete loss of SERT (SERT^−/−^) have high baseline anxiety associated behaviors, but the SERT^+/−^ rats have normal anxiety behaviors, which suggests that other risk factors such as acute or chronic stress may be needed to observe anxiety vulnerability^43,44^. In light of the SERT gene being a strong candidate gene associated with the severity of PA’s, but not being associated with increase panic symptoms post a CO_2_ challenge, we assessed baseline anxiety; innate panic responses to a CO_2_ challenge (as well as cellular responses in anxiety associated neural circuits); and fear conditioned responses in SERT^+/−^ and SERT^−/−^.

## Methods and materials

### Animals

SERT^−/−^ Wistar rats were purchased from genOway (Lyon, France) and were cross bred with wildtype SERT^+/+^ Wistar rats to produce SERT^+/−^ heterozygous Wistar Rats^42^. The behavior and gene array experiments were conducted on male 300–325 g rats, and the electrophysiological experiments male 150–200 g rats. All rats were housed individually in plastic cages under standard environmental conditions (22 °C; 12/12 light/dark cycle; lights on at 7:00 a.m.) for 7–10 days prior to the surgical manipulations. Food and water were provided ad libitum. All experiments were conducted in accordance with the *Guide for the Care and Use of Laboratory Animals*, Eighth Edition (Institute for Laboratory Animal Research, The National Academies Press, Washington, DC, 2011) and the guidelines of the IUPUI Institutional Animal Care and Use Committee.

### RT-PCR to assess SERT mRNA in dorsal/median raphe nucleus and Gene Array in BLA

Rats were rapidly anesthetized via IsoFlurane, decapitated, and brains were removed and frozen in isopentane chilled with dry ice, then stored at −80 ^°^C. In a RNase free environment, frozen brains were sliced coronally at 300 µm (coronal) using a cryostat, and sections were placed onto slides. The dorsal raphe nucleus (DRn) and median raphe nucleus (MRn) were dissected out of 3 adjacent sections and the BLA from 2 adjacent sections using a 1.0 mm Harris Micro-punch (Electron Microscopy Sciences) and RNA processing and RT-PCR analysis of custom TLDA were performed as previously described^45^. For DRn/MRn, comparative evaluation of quantitative real-time PCR was performed with validated Taqman probes (Assay ID: SERT; Rn00564737_m1 and beta actin; Rn00667869_m1, respectively, Applied Biosystems) using the Taqman gene expression master mix and 7900HT real-time PCR system (Applied Biosystems). Each sample was analyzed in triplicate, and beta actin was used as an internal standard. The BLA tissue was processed for serotonergic-related genes using the custom-designed TaqMan Low Density Array (TLDA), see supplemental table 1 for listing of genes. The gene expression panel was normalized using geNorm approach, which identified Gapdh, Ppia and Ppib (from the 9 endogenous control genes included in the custom TLDA) to be used for normalization, as previously described in ref. ^45^.

### The light and dark box test (LDBT), open field test (OFT), and elevated plus maze (EPM)

Details are provided in supplemental methods. All rats were handled 3 times a week for 3–5 min by experimenter prior to the day of behavioral assays. Briefly, the LDBT apparatus consisted of one lit compartment (50 lux) and one dark compartment, which were adjacent to each other with a small opening enabling the rat to transition between the boxes during 5 min test. The OFT arena had 4 squares forming the center; 12 squares forming the middle perimeter; and 20 squares forming the outer perimeter. On the test day, rats were placed in the center and the time spent in the different zones during the 5 min test. Both tests were video-recorded and scored using Anymaze video tracking software (Stoelting, Woods Dale, IL).

The elevated plus maze consisted of 2 long arms with no walls, an intersection, and 2 closed walls. On the test day, the animals were placed in the center area of the maze and allowed to explore the open and closed arms for 5 min. The total amount of time spent in each arm was recorded directly to the computer from the infrared beam breaks.

### Gas infusion procedure for in vivo respiration activity measures

All rats were handled 3 times a week for 3–5 min by experimenter prior to the day of gas infusions. Rats were placed in a clear Plexiglass® cylindrical plethysmograph chamber with atmospheric air infused at a flow rate of 2.8 l/min using a Vetamac flowmeter (Rossville, IN) until a steady baseline respiration rate was noted. All rats had infusions of the following: (1) 5 min infusion of atmospheric gas (<1% CO_2_, 21% O_2_, 79% N_2_) for baseline measurements, then (2) either the control gas or experimental normoxic, hypercarbic gas (7.5% CO_2_, 21% O_2_, 59% N_2_) for 5 min, and finally, (3) 5 min infusion of atmospheric gas. A pressure amplifier (model 24PC01SMT, Honeywell Sensor, Golden Valley, MN) was connected to a Powerlab data 8/35 acquisition system (ADInstruments, Colorado Springs, CO) and respiration rate was assessed by calculated using LabChart software (ADInstruments), and sighing was also visualized using Labchart software. A sigh was defined as an initial respiration that immediately precedes an additional second deeper inspiration. The sigh is followed by an expiration that is larger than usual, and is often accompanied by a post sigh apnea^46^. Supplemental Fig. 1 illustrates the changes in the concentrations of CO_2_ and O_2_ that occur prior to, during and at offset of gas infusion.

Ninety min following the initiation of treatment, rats were anesthetized with an overdose of isoflurane then perfused transcardially with 0.05 M phosphate buffered saline (PBS; 250 ml), followed by PBS containing 4% paraformaldehyde (PFA) and 3% sucrose and the brains were removed and processed for immunohistochemistry as described in detail previously^47^.

### Immunohistochemistry procedures for c-Fos and SERT

Free-floating sections were washed in 0.05 M PBS for 10 min × 3 between initial incubation in 1% H_2_O_2_ in PBS for 20 min. Immunostaining for SERT protein and c-Fos protein was accomplished with sequential immunohistochemical procedures using (1) primary antibodies directed against SERT (rabbit anti-SERT polyclonal antibody, Cat. no. 24330, Immunostar, diluted 1,1000) and c-Fos (rabbit anti-c-Fos polyclonal antibody, Cat. no. sc-52, Ab-5, Santa Cruz Biotech., Santa Cruz, CA, USA; diluted 1:10,000) which were incubated on orbital shaker 12–14 h then washed in PBS prior to and in between 2 h incubations in: (1) biotinylated secondary antibodies; (2) avidin biotin complex kit; and (3) chromogen kit as described previously^47^.

### Densitometry of SERT-ir in dorsal raphe nucleus (DRn) and Counting of c-Fos-ir cells

Selection of anatomical levels for analysis of SERT immunostaining in the DRn and c-Fos-immunostaining in anxiety circuits was conducted with reference to illustrations from a rat brain stereotaxic atlas^48^. Photomicrographs of the SERT immunostaining in the DRn were taken at ×400 magnification using a DMLB Leica binocular brightfield microscope at 3 Bregma levels [i.e., −7.4, −7.5, −7.6 mm from Bregma] for each rat. Densitometry analyses were done on grayscale inverted photographs using Adobe Photoshop version 16 for each Bregma level for each rat and combined prior to analyses. The WT mean represented 100% which was compared to SERT^+/−^ and SERT^−/−^ values.

The numbers of c-Fos-ir cells were counted in the entire field of view at ×400 magnification in brain regions implicated in innate panic responses [i.e., the dorsomedial hypothalamic nucleus (DMN) at −2.80 mm Bregma and dorsolateral periaqueductal gray (DLPAG) at −8.00 mm Bregma] and conditioned fear responses [i.e., central amygdaloid nucleus (CeA); basolateral amygdaloid nucleus (BLA), basolateral amygdaloid nucleus, ventral part (BLV); lateral amygdaloid nucleus (LA); and medial amygdaloid nucleus (MeA) at −2.80 mm Bregma]. All cell counts were done by an observer that was blind to the experimental treatment of each animal.

### Photography

Photomicrographs were obtained the Leica DMLB microscope connected to a Leica DFC 300 digital camera and Leica Applications Suite 4.1 for Windows.

### Fear conditioning protocol

All rats were handled 3 times a week for 3–5 min by experimenter prior to the day of behavioral assays. The fear-conditioning chamber has a grid floor composed of 6 stainless steel rods connect to a shock generator (Kinder Scientific, Poway, CA, USA). The fear conditioning protocol was 4 days long. On day 1, rats were placed in the conditioning chamber and allowed to habituate for 10 min. On day 2, test day 1, the rats were placed back in the conditioning chamber and was exposed to 5 tone + shock pairings, with a 120 s inter-trial interval with a tone conditioned stimuli (CS: 80 dB, 20 s) co-terminating with a single shock unconditioned stimuli (UC: 0.80 mA, 500 ms). On day 4, test day 2, rats underwent an extinction paradigm where they were presented with 20 tone CS only with a 120 s inter-trial interval. All sessions were video-recorded and the total time spent freezing during the tones on all 2 test days was scored blind by the investigator Stephanie Fitz.

### Injections of 5-HT_1A_ receptor agonist into BLA prior to fear acquisition and extinction of SERT^+/−^ rats

All rats were handled 3 times a week for 3–5 min by experimenter prior to the day of behavioral assays. SERT^+/−^ rats (*n* = 9) were anaesthetized by placing them in a closed Plexiglas® box that was connected to an isoflurane system (MGX Research Machine; Vetamic, Rossville IN, USA) and then with a nose cone connected to the same system during the stereotaxic surgery. Rats were placed into an ultra-precise stereotaxic instrument (Kopf Instruments, Tujunga, CA, USA) with the incisor bar set at –3.3 mm and nose cone connected to the same system during the surgery. Two stainless steel guide cannulae (26 gauge, Plastics One, Roanoke, VA) were situated into guide cannulae holders fixed onto the stereotaxic arms. The 33 gauge injector was lowered into position of the BLA using coordinates (anterior, –2.1 mm; lateral, ±5.0 mm; ventral, –8.5 mm) according to a standard stereotaxic atlas of the adult rat brain^48^. The guide cannulae were secured into place using three 2.4 mm screws anchored into the skull along with cranioplastic cement. Following placement of dummy cannulae into the guide cannulae, rats were removed from the stereotaxic apparatus and allowed to recover for 72 h prior to experimental testing. SERT^+/−^ rats received bilateral 200 nL injections of either artificial cerebrospinal fluid (aCSF, *n* = 5) or 20 mM 8-Hydroxy-2-(dipropylamino)tetralin hydrobromide (8-OH-DPAT, a 5-HT_1A_ agonist, Sigma, *n* = 4) 20 min prior to acquisition and recall/extinction at a rate of 100 nL/min using 10-μl Hamilton syringes attached to an infusion pump (Harvard Apparatus, model PHD 2000) and subsequently connected to the injection cannulae via polyethylene (PE 50) tubing (Fisher Scientific, Pittsburg, PA). Once the injection cannulae were securely placed into the rat, the infusion pump was turned on and set to automatically deliver 100 nL/site over 2 min. Following the injection, the cannulae remained in place for an additional min before being removed. Smooth flow of the solutions via the tip of the injection cannulae was verified before and after each injection to ensure proper drug delivery.

### Electrophysiology

Once anesthetized, brains were quickly dissected, and 350-μm coronal slices were obtained as described earlier^49^. Briefly, slices were incubated in an oxygenated (95% O_2_/5% CO_2_ mixture) artificial cerebrospinal fluid (ACSF) of the following composition (in mM): 130 NaCl, 3.5 KCl, 1.1 KH_2_PO_4_, 1.3 MgCl_2_, 2.5 CaCl_2_, 10 glucose, 30 NaHCO_3_ at room temperature for ~1 h before the recording. Slices containing the BLA were then transferred to a recording chamber mounted on the stage of the upright Nikon E600FN microscope (Nikon Instruments Inc., Melville, NY) and perfused at rate of 2–3 ml/min with ACSF heated to 30 °C. Whole-cell patch clamp recordings were obtained using standard techniques with borosilicate glass electrodes (resistance 3–5 MΩ, WPI, Sarasota, FL) filled with a potassium gluconate-based solution of the subsequent composition (in mM): 140 potassium gluconate, 2 KCl, 3 MgCl_2_, 10 HEPES, 5 phosphocreatine, 2 potassium-ATP, 0.2 sodium-GTP) adjusted to pH 7.3 with KOH, and having an osmolarity of 285–295 mOsm. Whole-cell access resistance varied between 5 to 20 MΩ and was examined during each experiment; a change of less than 15% was acceptable. The data were obtained only from pyramidal neurons located in BLA. Whole-cell recordings were made with a Multiclamp 700B amplifier (Molecular Devices, Sunnyvale, CA) using the pClamp 10.3 software and a Digidata 1322 A interface (Molecular Devices, Sunnyvale, CA).

### Statistical analyses

The following dependent variables were analyzed using an ANOVA with genotype as the main independent variable (i.e., light dark box test, open field and elevated plus maze, sighs, c-Fos-ir cells, SERT-ir densitometry, and electrophysiology data). Respiratory responses over time and fear conditioned freezing behavior was analyzed using a one way ANOVA with repeated measures with *genotype* as independent variable and time as the repeated measures. In the presence of significant effects, between and within subjects post-hoc tests were conducted using a Fisher’s LSD or Dunnett’s post-hoc test, respectively. Statistical significance was accepted with *p* ≤ 0.05. All statistical analyses were done with SPSS 25 and Graphpad Prizm 7.0; and all graphs were generated using Graphpad Prizm 7.0 for Windows. Figureplates were made with CorelDraw X for Windows.

## Results

### Genotyping and SERT mRNA expression in DRn/MRn and SERT immunohistochemistry in the DRn

**Fig. 1:**
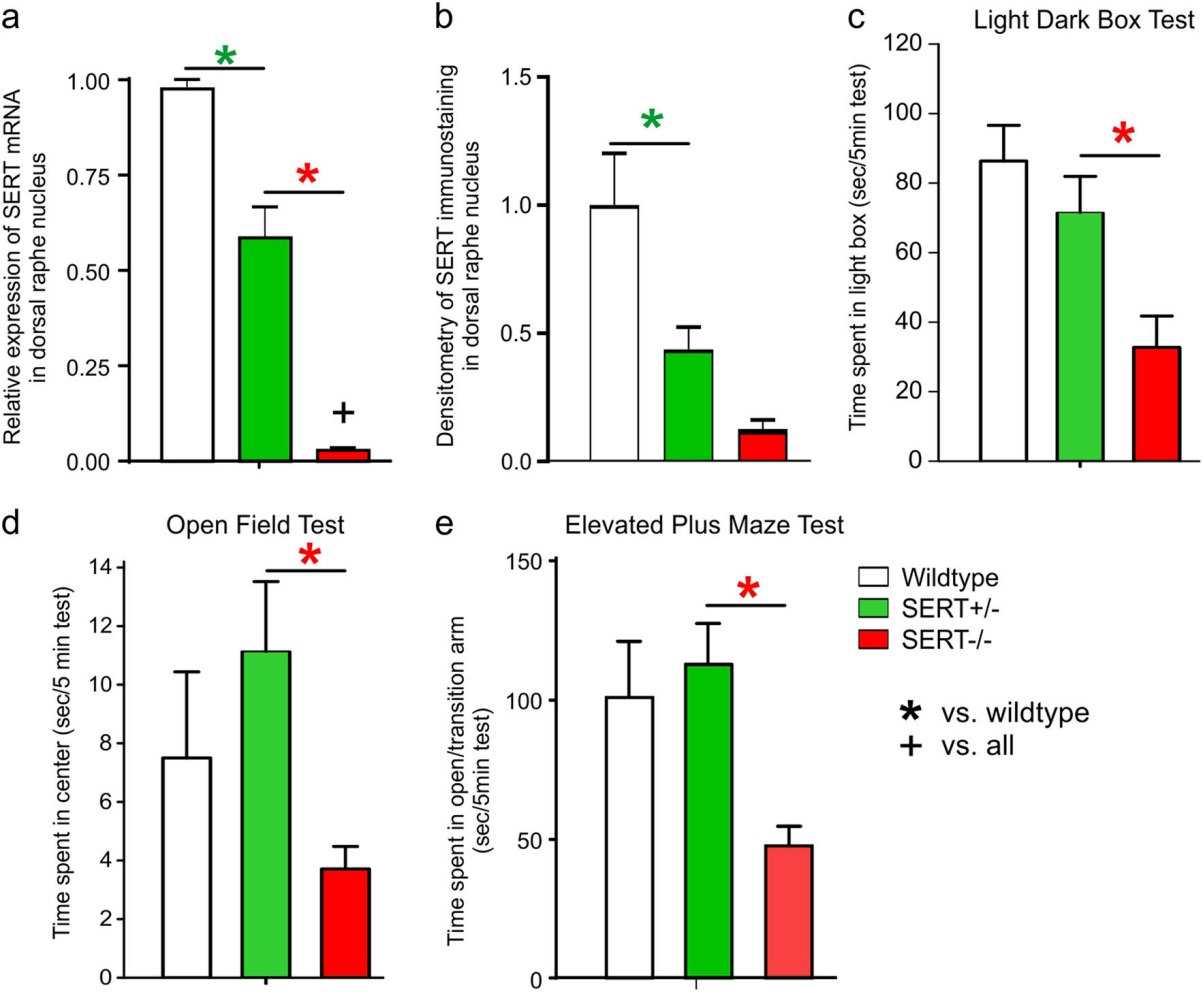
SERT mRNA in DRn/MRn and SERT immunohistochemistry in DRn. Bar graphs represent SERT (**a**) mRNA expression in the combined dorsal/median raphe nuclei; (**b**) densitometry analyses of SERT immunohistochemistry in the dorsal raphe nuclei and (**c**–**e**) baseline anxiety-associated behaviors between wildtype, SERT^+/−^ and SERT^−/−^ genotypes: (**c**) duration of time in light box, compared to dark box; (**d**) time spent in center of an open field; and (**e**) time spent on the combined open arm and transition zone. Bars represent mean and error bars the standard error of the mean (SEM). Asterisks(*) indicates posthoc significance with a Fisher’s LSD, *p* < 0.05

In order to verify the diminished SERT transcriptional efficiency of SERT^+/−^ and SERT^−/−^ rats, we measured SERT mRNA in micropunched tissue containing the DRn/MRn region, which revealed that, compared to wildtype SERT^+/+^ controls (*n* = 3), SERT^−/+^ rats (*n* = 5) had approximately a 40% reduction SERT mRNA and SERT mRNA levels were at the limit of detection for SERT^−/−^ rats (*n* = 6, F_(2,11)_ = 83.1, *p* < 0.001, Fig. 1a). We also assessed SERT protein expression with a densitometry analysis of SERT immunostaining in the DRn where compared to WT rats (*n* = 6), SERT^+/−^ (*n* = 6) and SERT^−/−^ (*n* = 6) rats had ~57% and ~88% reduction in SERT immunostaining, respectively [F_(2,15)_ = 11.9, *p* = 0.0008, Fig. 1b], which is consistent with a previous characterization^42^.

### Light dark box test, open-field behavior and elevated plus maze

**Fig. 2:**
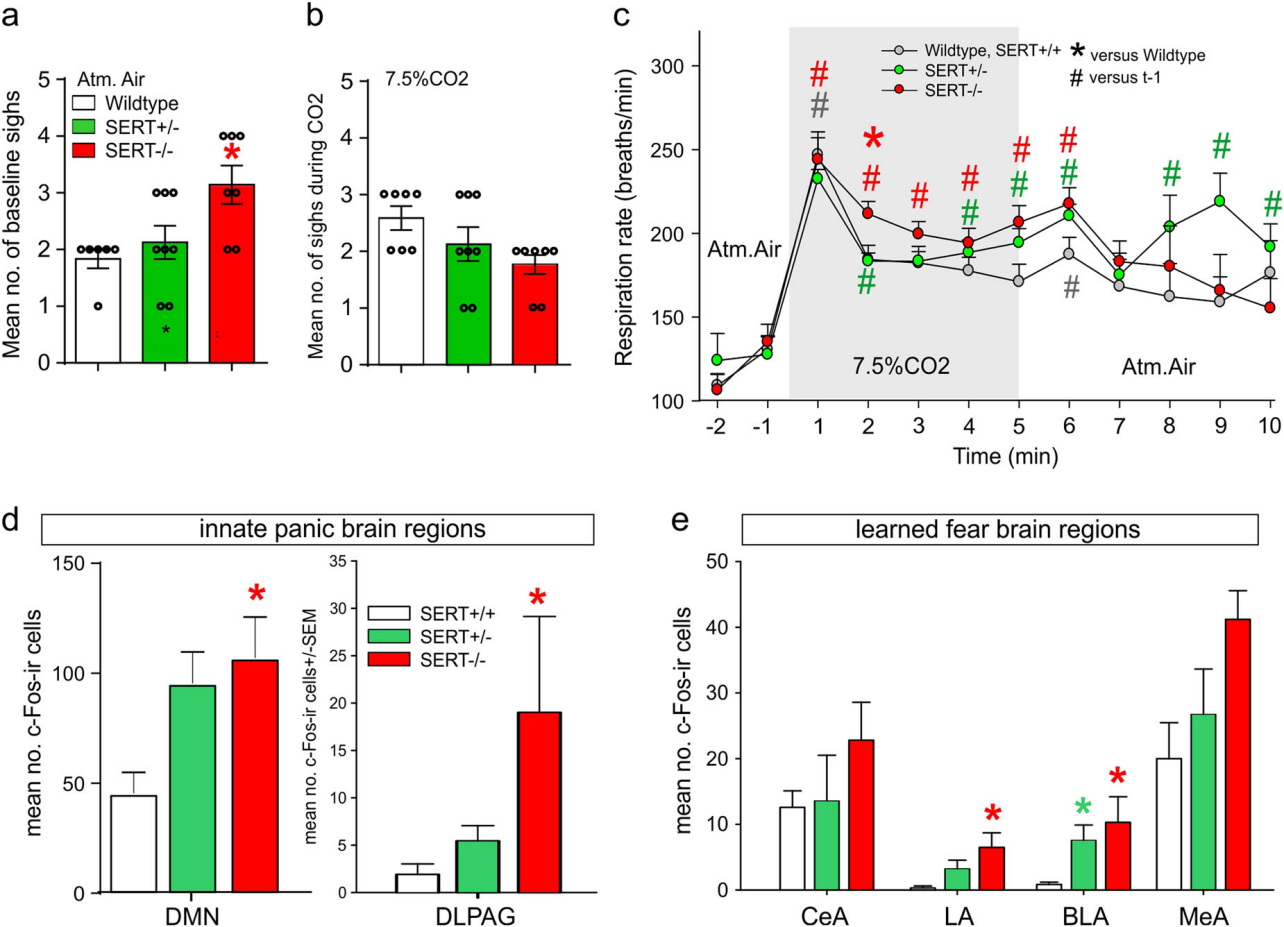
Respiratory and cellular c-Fos responses to CO_2_ challenge. **a**, **b** Represents bar graphs of the no. of sighs at baseline and during infusion of 7.5%CO_2_ (normoxic). **c** Line graph represents the respiratory rate responses (breaths/min) for wildtype, SERT^+/−^ and SER^−/−^ rats during atmospheric air infusions (non-shaded regions) and during hypercapnic gas (7.5% CO_2_, normoxic air, shaded region) infusions. Asterisks(*) indicates significant between subjects effects with a Fishers LSD posthoc test protected by an ANOVA, and hastag(#) indicates significant within subject time effects with a Dunnett’s test protected by a repeated measures one way ANOVA. **d**, **e** bar graph represents ex vivo cellular responses (evidenced by numbers of cells with nuclear expression of c-Fos protein immunostaining) in the dorsomedial hypothalamic nucleus (DMN), dorsolateral periaqueductal gray (DLPAG) and amygdala nuclei following exposure to 7.5%CO2 in wildtype, SERT^+/−^ and SERT^−/−^ rats. Error bars represent the SEM

Baseline assessment of innate anxiety-associated behaviors revealed that, compared to both wildtype SERT^+/+^ rats (*n* = 8) and SERT^+/−^ rats (*n* = 13), only SERT^−/−^ rats (*n* = 11) displayed reduced time spent: (1) in the light box (F_(2,29)_ = 5.7, *p* = 0.008, Fig. 1c); (2) in the center zone of open field (F_(2,29)_ = 4.0, *p* = 0.029, Fig. 1d); or in the open arm + transition zone of the elevated plus maze (F_(2,29)_ = 4.5, *p* = 0.019, Fig. 1e). Compared to WT rats, SERT^−/−^ rats also had less transitions between light and dark box in LDBT (F_(2,29)_ = 6.0, *p* = 0.0065, data not shown) and the number of entries into the open arm of EPM approached significance as being reduced (F_(2,29)_ = 3.3, *p* = 0.0511, data not shown). There was no difference in center time entries in OFT (F_(2,29)_ = 0.7, *p* = 0.515, data not shown).

### Respiratory responses to atmospheric versus CO_2_ challenge

Assessments of respiratory activity in conscious freely moving rats revealed that, compared to wildtype SERT^+/+^ (*n* = 6, one outlier detected with a Grubb’s test, *p* < 0.05) and SERT^+/−^ (*n* = 8), SERT^−/−^ rats (*n* = 7) had an increased no. of sighs (F_(2,18)_ = 5.4, 0.014), compared to WT rats during atmospheric control gas infusions (Fig. 2a, see also supplemental Fig. 2a,b). Although there was no difference in sigh frequency during the CO_2_ challenge (F_(2,19)_ = 3.1, *p* = 0.071, Fig. 2b), in a followup experiment there was a significant *genotype* *×* *time interaction* detected on respiration rate during the CO_2_ challenge (F_(22,308)_ = 1.6, *p* = 0.047, Fig. 2c, see also supplemental Fig. 2c). Specifically, posthoc analyses revealed that SERT^−/−^ rats (*n* = 11), but not SERT^+/−^ rats (*n* = 13), displayed a higher respiration rate during CO_2_ challenge, compared to wildtype SERT^+/+^ controls (*n* = 7).

### Cellular responses in innate panic and learned fear network following a 7.5%CO_2_ challenge

Briefly exposing rats to 7.5% hypercarbic gas increased the number of c-Fos-ir cells in brain regions implicated in innate panic responses [i.e., DMN: Kuskall Wallis ANOVA = 5.9, *p* = 0.05) and also in the DLPAG: Kruskall Wallis ANOVA = 7.7, *p* = 0.02) in only the SERT^−/−^ rats (Fig. 2d)]. Within learned fear circuits, exposing rats to hypercarbic gas increased numbers of c-Fos-ir cells in the LA (Kruskall–Wallis ANOVA = 8.7, *p* = 0.01) of only SERT^−/−^ rats, but for the BLA this occurred in both SERT^+/−^ and SERT^−/−^ rats (Kruskall–Wallis ANOVA = 9.5, *p* = 0.001, Fig. 2e).

### Fear conditioning behaviors

**Fig. 3:**
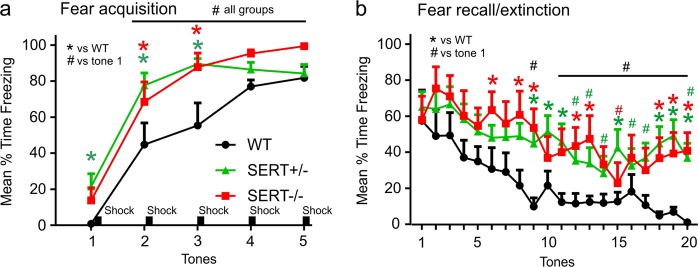
Fear conditioned behaviors. Line graph represents the mean duration of freezing of wildtype (*n* = 6), SERT^+/−^ (*n* = 12) and SERT^−/−^ (*n* = 8) rats during a presentation of an acoustic tone on the following days: (**a**) acquisition of conditioning with 5 tones followed by 5 mild shocks on day 1; (**b**) evidence of consolidation on day 2 with initial tone and no shock and extinction learning with subsequent tones and no shock. Asterisks(*) and hastag(#) indicates significant between subjects effects with a Fishers LSD posthoc test or within subject differences over time with a Dunnett’s test (versus tone one, baseline) protected by a repeated measures one way ANOVA. Error bars represent the SEM

On acquisition day all rats displayed increased freezing over time with repeated pairings of the conditioned stimulus (tone) with the unconditioned stimulus (shock). However, a more rapid acquisition was detected in both the SERT^−/−^ (*n* = 8) and SERT^+/−^ (*n* = 12) rats, compared to wildtype SERT^+/+^ controls (*n* = 6, not *genotype* × *tone interaction* F_(8,92)_ = 2.0, *p* = 0.048, Fig. 3a). On test day 2 (evidence of consolidation), there was no significant *genotype* *×* *tone interaction, F*_(34,437) = _0.8, *p* = 0.80 but there was an overall *genotype effect*, F_(2,23)_ = 3.6, *p* = 0.044 detected. Between subjects posthoc analyses revealed that there was no difference in recall between groups on tone 1 with no shock. However, the SERT^−/−^ and SERT^+/−^ rats showed higher freezing on subsequent tones and had delayed extinction learning (Fig. 3b).

### Intrinsic and network properties of BLA principal neurons of SERT^+/−^, SERT^−/−^, and WT rats

**Fig. 4:**
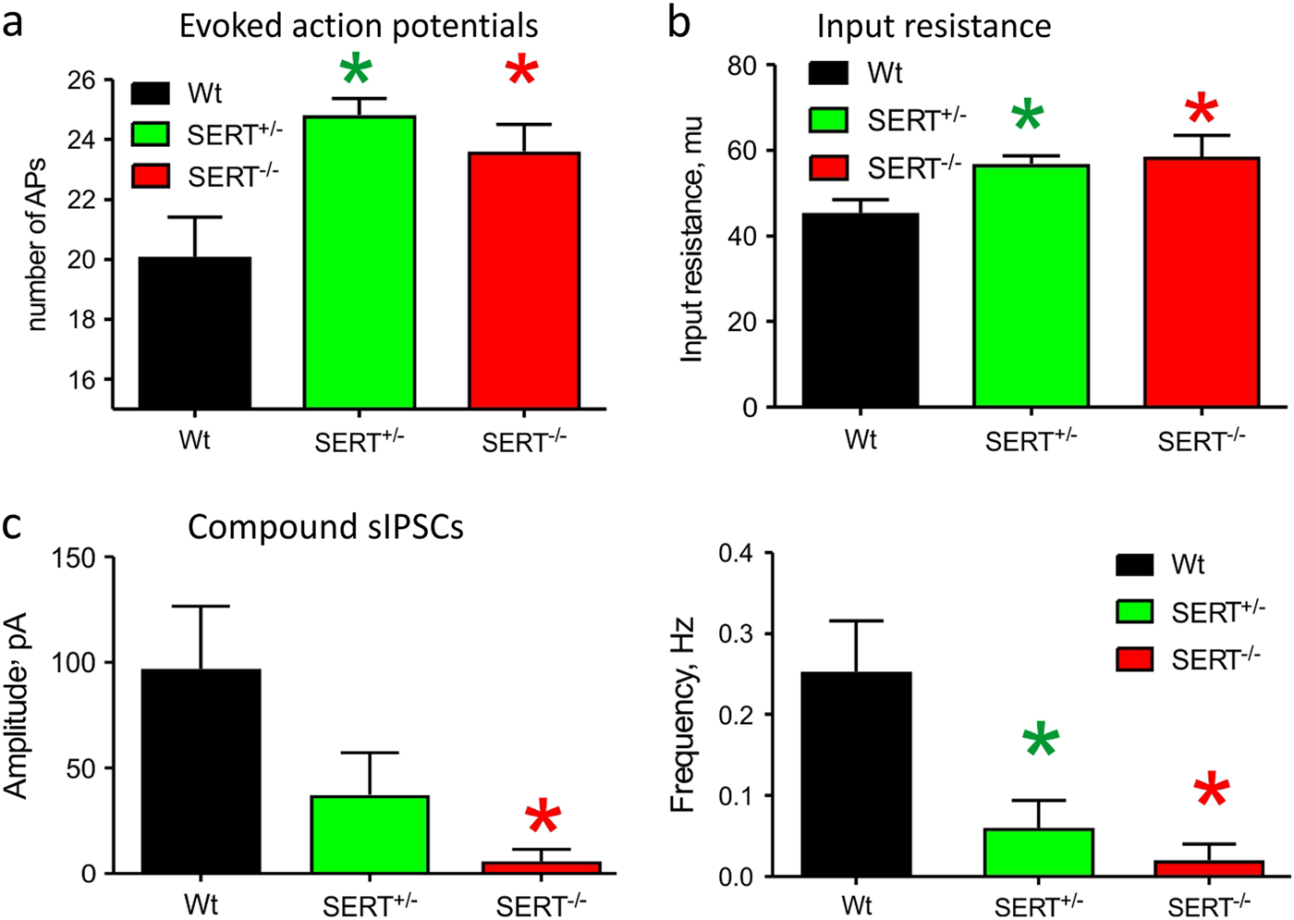
Intrinsic and network properties of BLA principal neurons. **a** bar graph summarizing the number of evoked action potentials of WT, SERT^+/−^ and SERT^−/−^ rats; (**b**) Bar graph represents input resistance of the BLA principal neurons of wildtype (*n* = 12), SERT^+/−^ (*n* = 11) and SERT^−/−^ (*n* = 10) rats; (**d**) representative spontaneous activity recordings in voltage-clamp mode from BLA neurons of WT, SERT^+/−^ and SERT^−/−^ rats, (**e**) bar graphs summarizing amplitude and frequency of spontaneous compound IPSCs of WT, SERT^+/−^ and SERT^−/−^ rats; (**f**) bar graphs summarizing amplitude and frequency of sEPSCs of WT, SERT^+/−^, and SERT^−/−^ rats. Error bars represent the SEM

We calculated number of action potentials induced by the injected current of the same value. The BLA neurons from SERT^+/−^ and SERT^−/−^ animals fired significantly higher number of evoked action potentials compared to the WT rats [F_(2,30)_ = 6.04, *p* = 0.006, Fig. 4a, see also supplemental Fig. 3a]. Additionally, we also observed significantly higher membrane input resistance between WT (*n* = 12), SERT^+/−^ (*n* = 11) and SERT^−/−^ (*n* = 10) groups [F_(2,30)_ = 4.56, *p* = 0.02, Fig. 4b]. Last, we also looked at BLA network properties by measuring amplitude and frequency of spontaneous excitatory (EPSCs) and inhibitory currents (IPSCs). We noticed a significant reduction of amplitude and frequency of spontaneous compound IPSCs in SERT^+/−^ and SERT^−/−^ rats compared to WT [amplitude: F_(2,30)_ = 4.41, *p* = 0.021; frequency: F_(2,30)_ = 7.77, *p* = 0.002, Fig. 4c, see also supplemental Fig. 3b]. No changes in amplitude and frequency of sEPSCs were observed [amplitude: F_(2,29)_ = 0.13, *p* = 0.883; frequency: F(2,29) = 0,11, *p* = 0.896, supplemental Fig. 3b, c).

### Custom serotonin-related gene TLDA

**Fig. 5:**
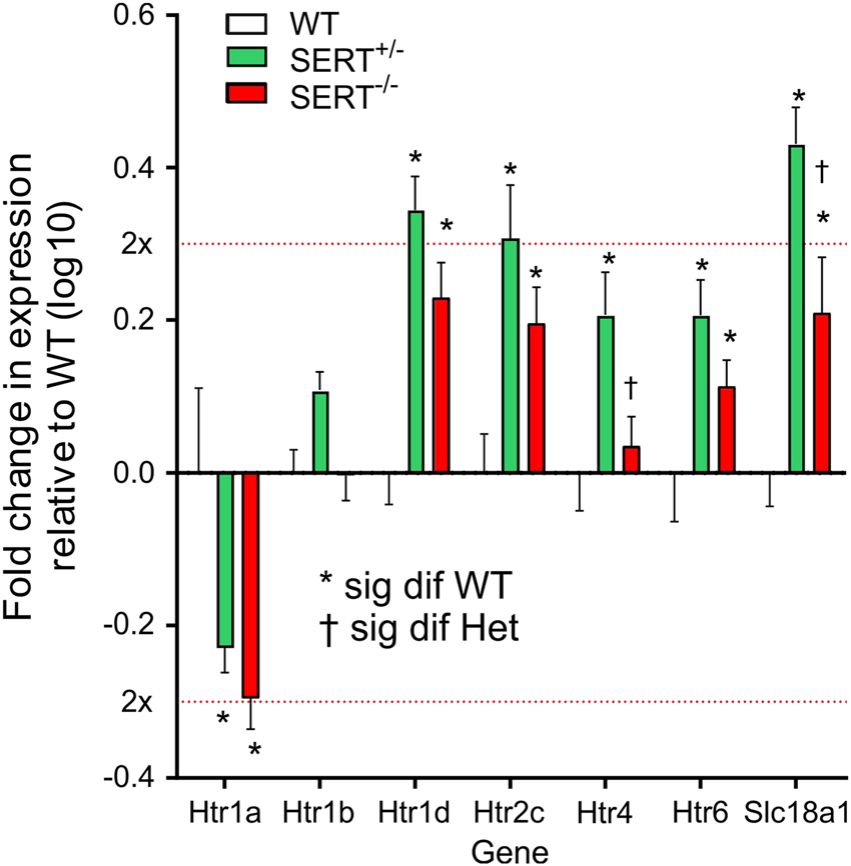
Expression profile of serotonin-related genes in the BLA by genotype. Presented here are the mean (±SEM) expression, relative to WT, of serotonin receptor genes and one vesicular monoamine transporter 1 gene that were differentially expressed between genotypes. Dotted red lines represent 2 fold increase or decrease in relative expression. Asterisks(*) indicates significantly deferent from WT (Tukey’s *p* ≤ 0.022) and † indicates significantly different from SERT^+/−^ (Tukey’s *p* = 0.0082). Error bars represent the SEM

Among the twenty-one serotonin related genes (see Suppl. Table 1) that were assessed within the BLA of WT, SERT^+/−^, and SERT^−/−^ (n = 5/ genotype) expression of four genes, Htr2b, Htr3b, Htr5b and Slc6a4 (SERT) were below detection limits (Ct > 34) in the BLA and were removed from analysis. The relative expression of remaining 17 genes had significant resulted in significant main effects of Genotype, Gene and Genotype *×* Gene interaction (2way ANOVA F_2,170_ = 4.925, *P* = 0.0083; F_16,170_ = 4.516, *P* < 0.0001; and F_23,170_ = 1.641, *P* = 0.0241, respectively). Seven genes were differentially express between genotypes (Fig. 5 and Suppl. Table 1). Expression of the 5-HT_1A_ receptor gene (Htr1a) was significantly reduced compared to WT in both SERT^+/−^ and SERT^−/−^ groups (Tukey’s p = 0.0099 and 0.0003, respectively). SERT^+/−^ and SERT^−/−^ groups had significantly increased expression, compared to WT, of Htr1d (5-HT_1d_ receptor), Htr2c (5-HT_2c_ receptor) and Slc18a1 (vesicular monoamine transporter) genes (Tukey’s *p* ≤ 0.022). Additionally, expression of Htr4 (5-HT_4_ receptor) and Htr6 (5-HT6 receptor) genes were both significantly increased in the SERT^+/−^ group compared to WT (Tukey’s *p* = 0.0215 and 0.022, respectively).

### Injections of 5-HT_1A_ receptor agonist into BLA prior to fear acquisition and extinction of SERT^+/−^ rats

**Fig. 6:**
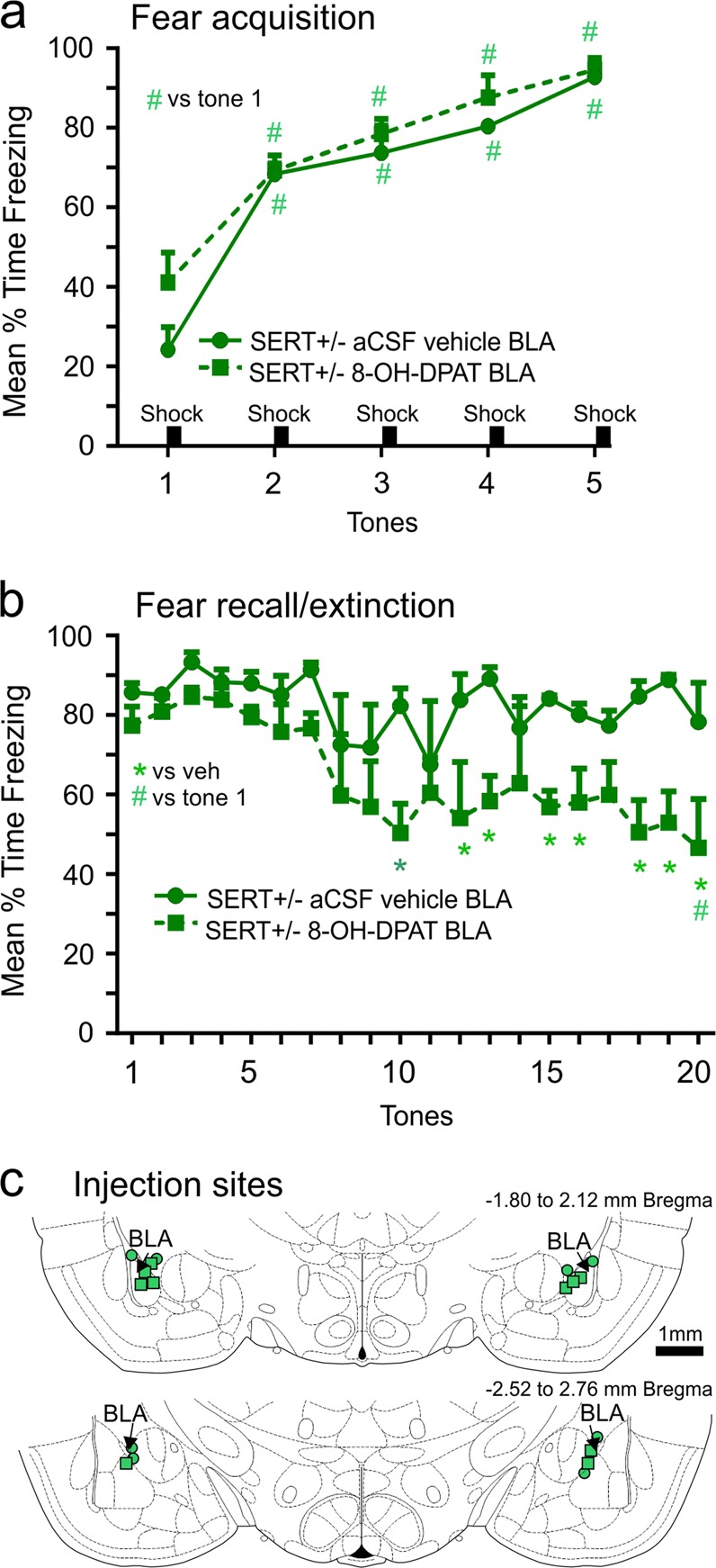
Fear conditioned behaviors in SERT^+/-^ rats following 5-HT_1A_ receptor agonist injections in the BLA. Line graph represents the mean duration of freezing of SERT^+/−^ (*n* = 4) rats with bilateral injections of either artificial cerebral spinal fluid vehicle (*n* = 5) or the 5-HT_1A_ receptor agonist 8-OH-DPAT into the BLA prior to (**a**) acquisition of conditioning with 5 tones followed by 5 mild shocks on day 1; (**b**) evidence of consolidation on day 2 with initial tone and no shock and extinction learning with subsequent tones and no shock. * and # indicates significant between subjects effects with a Fishers LSD posthoc test or within subject differences over time with a Dunnett’s test (versus tone one, baseline) protected by a repeated measures one way ANOVA. (**c**) Coronal illustration of a Standard stereotaxic atlas of the rat brain with green circle and green square symbols indicating injection cannula for SERT^+/−^ rats injected with either vehicle or 8-OH-DPAT, respectively. Error bars represent the SEM

On acquisition day there was no difference in freezing acquisition between SERT^+/−^ rats with bilateral injections of artificial cerebrospinal fluid vehicle (*n* = 5) or with 8-OH-DPAT (*n* = 4) into the BLA (Fig. 6a, overall tone effect detected F_(4,28)_ = 46.0, *p* < 0.0001, but no overall *treatment effect* or *treatment* × *tone interaction* F_(4,28)_ = 0.9, *p* = 0.492). On test day 2, there was no significant *treatment* × *tone interaction*, but there was an overall *treatment effect*, F_(1,7)_ = 8,5, *p* = 0.022 detected. Between subjects posthoc analyses revealed that there was no difference in recall between groups on initial tones with no shock. However, the SERT^+/−^ rats pretreated with 8-OH-DPAT into the BLA displayed decreased freezing on subsequent tones (Fig. 6b). Histology verified that all injections were within BLA region (Fig. 6c).

## Discussion

Here we first confirmed that that there was reduced transcription of SERT mRNA and protein expression within the DRn and MRN of SERT^+/−^ rats and at the limit of detection in SERT^−/−^ rats, then also confirmed that SERT^−/−^ rats, but not SERT^+/−^ rats, had high baseline anxiety associated behaviors, which are both consistent with previous reports^42–44^. We then exposed SERT^+/−^ and SERT^−/−^ rats to a viscerosensory challenge [i.e., 5 min 7.5% hypercapnic gas exposure] that induces PA’s in the majority of humans with PD, but not healthy controls^50–52^. Only SERT^−/−^ rats showed evidence of panic vulnerability which was evidenced by increased baseline sighing and heightened tachypnea to the hypercapnic challenge, which was also associated with increased cellular responses in two brain regions [i.e., the DPAG and DMH] where PA’s can be induced in human by electrical stimulation^53–55^ and where panic associated behaviors and physiological responses can be induced in rats following pharmacological disinhibition^56–59^. The hypothesis that the high baseline sighing and increased respiratory rate responses to 7.5%CO_2_ challenge in only SERT^−/−^ rats potentially indicates panic vulnerability is consistent with clinical data. Specifically, a meta-analysis of baseline respiratory parameters in PD determined that PD was associated with higher baseline rate of sighing which may a hyper responsive reaction to increases in acidosis, a suffocation signal^60^. This is also consistent with symptoms such as dyspnea, feeling out of breath, or suffocating during PAs, and that PA’s can be reliably induced in humans with PD with 5-7% hypercapnic gas exposure^5–7^. The lack of differences in sighing post CO_2_ is most likely due to the rapid increase in respiration rate in all rats, but that was also hyperactive in SERT^−/−^ rats. These SERT^+/−^ data are also consistent with humans with the SERT s-allele polymorphism not displaying increased panic symptoms following a panicogenic CO_2_ challenge^41^.

Assessment of cellular responses within the amygdala post CO_2_ revealed that both SERT^-/+^ and SERT^−/−^ rats had significant increases within the BLA, compared to WT rats. This was an interesting observation since previous studies have shown that even high concentrations of 20%CO_2_ which induces panic associated behaviors and physiology in control rats^61–63^, does not increase c-Fos responses in the amygdala^62^. Because we have noted an increase in number of c-Fos positive cells in the BLA which plays a critical role in fear conditioned responses [see reviews^35,36^], we next assessed cue induced fear conditioned responses. Compared to WT rats, both SERT^+/−^ and SERT^−/−^ rats displayed similar increases in the acquisition of cue induced freezing and resistant fear extinction 48 h later. These SERT^+/−^ data are consistent with humans with the SERT s-allele polymorphism having enhanced fear conditioned responses^25,26^ and being associated with a higher risk of PTSD in humans exposed to high trauma^27^. These data suggest that although SERT^+/−^ rats are not vulnerable to CO_2_ induced panic, they are prone to acquiring phobias which is a conditioned avoidance response that occurs when people with PD begin to fear situations/stimuli that are associated with PA’s.

Since the SERT s-allele polymorphism is also associated with enhanced baseline amygdala activity and reactivity to fearful stimuli;^37,38^ we next assessed the basic and network properties of BLA principal neurons. Here both SERT^+/−^ and SERT^−/−^ rats show a reduction in the inhibition within the BLA which was also associated with enhanced evoked action potentials. Overall, these data suggest that disruption of SERT transcription in both SERT^+/−^ and SERT^−/−^ rats induces a prolonged presence of serotonin in the synapses and the extrasynaptic space which disrupts the function of the local amygdala GABAergic network, which may also contribute in the overall increase of the excitability of the BLA neurons. Extracellular levels of 5-HT increase rapidly within the BLA during fear conditioning^64^ and high concentrations or prolonged application of 5-HT reduce the frequency and amplitude of spontaneous IPSP/Cs^65^. Prolonged increases in extracellular 5-HT also occur in response to inescapable stress^66^, which may contribute to a net loss of local GABA inhibition, as seen here, and subsequent increase in excitation of glutamatergic projection neurons following evoked responses or in response to threat. In order to gain more insights into molecular serotonergic network changes in the BLA of each genotype we next assessed local expression of serotonergic associated genes. Here we observed similar changes in gene expression in both SERT^+/−^ and SERT^−/−^ rats, which correlated with abnormalities in fear condition behaviors, but not with baseline anxiety or panic vulnerability to CO_2_ challenge. Specifically, these genotypes were associated with an approximately 2 fold reduction in the 5-HT1A receptor (*Htr1a*), and an ~ 2 fold increase in the 5-HT2C (*Htr2c*) and 5HT1d (*Htr1d*) receptor. Within the amygdala 5-HT receptor subtypes mediate both excitatory and inhibitory actions on GABAergic interneurons and glutamatergic projection neurons^67^. Glutamatergic neurons in the BLA express both 5-HT1A and 5-HT2C receptors^67,68^, which, respectively, are inhibitory and excitatory metabotropic receptors. In regards to fear conditioning, 5-HT1A agonist injections into the BLA region will reduce fear conditioned freezing responses in rats^69^ and in hamsters^70^, which is consistent with our data where injecting a 5-HT1A agonist into the BLA of a SERT^+/−^ enhanced fear extinction. Conversely, BLA injections of a selective 5-HT2C antagonist can attenuate fear conditioned behaviors^71^. Thus the decreased expression of the 5-HT1A receptor and increased expression of the 5-HT2C receptor could shift the effects of 5-HT release onto glutamatergic neurons to being more excitatory and thus enhance fear acquisition. During fear extinction the infralimbic region of the prefrontal cortex plays a critical role in enhancing fear extinction partially through inhibition of the BLA glutamatergic neurons^72–75^. Thus, chronic excitatory effects of 5-HT onto glutamatergic BLA neurons of SERT^+/−^ and SERT^−/−^ rats may interfere with inhibitory influence to induce deficits in safety learning. Overall, this expression profile could partially explain enhanced evoked action potentials of glutamatergic neurons in the BLA of SERT^+/−^ and SERT^−/−^ rats.

## Conclusions

Our data are consistent with previous experiments where depleting 5-HT levels in the BLA region diminishes fear conditioned behaviors^76^. Although only the SERT^−/−^ rats showed a panic vulnerability to CO_2_, and the SERT s-allele polymorphism is not associated with increases in PA/PD incidence, it is interesting that reduced 5-HT1A binding within the amygdala of humans with PD^77,78^ which could explain an increased incidence of expected PAs and high phobia comorbidity and also that PAs are more severe in PD humans with the SERT s-allele polymorphism^29^. Overall, these data are also supportive of the hypothesis that increased 5-HT activity within the amygdala may be contributing to the pathophysiology of expected PA’s, phobia vulnerability and PTSD^64,79^.

### Supplemental methods and results

#### RT-PCR to assess SERT mRNA in dorsal/median raphe nucleus and gene array in BLA

Rats were rapidly anesthetized via IsoFlurane, decapitated, and brains were removed and frozen in isopentane chilled with dry ice, then stored at −80^ °^C. In a RNase free environment, frozen brains were sliced coronally at 300 µm (coronal), using a cryostat, and sections were placed onto slides. The dorsal raphe nucleus (DRn) and median raphe nucleus (MRn) were dissected out of 3 adjacent 300 µm sections [from −7.0 to −8.0 mm (DRn) and −7.5 to −8.5 mm from bregma using a 1.0 mm Harris Micro-punch (Electron Microscopy Sciences) and were placed into 75 µl of SurePrep^TM^ TrueTotal^TM^ RNA purification buffer/kit to isolate RNA (Fisher Scientific). Samples were vortexed and Total RNA was determined using Nanodrop 1000. Extracted RNA was then reverse transcribed using the GeneAmp Gold RNA PCR kit (Applied Biosystems) at the following reaction conditions: 2.5 μM Oligo-dT primer, 2.5 mM magnesium, 250 mM of each deoxynucleotide triphosphate, 0.5 U/ml of RNase inhibitor and final concentration of 0.75 U/μl of MuLV reverse transcriptase. The reverse transcription conditions were 10 min at room temp, 15 min at 42° C, 10 min at 68° C and 5 min at 95°C and produced ~25 μl of product. Comparative evaluation of quantitative real-time PCR was performed with validated Taqman probes (Assay ID: SERT; Rn00564737_m1 and beta actin; Rn00667869_m1, respectively, Applied Biosystems) using the Taqman gene expression master mix and 7900HT real-time PCR system (Applied Biosystems). Each sample was analyzed in triplicate, and beta actin was used as an internal standard.

The BLA region was bilaterally dissected from two consecutive 300 µm coronal sections and tissue was processed for serotonergic-related genes using the custom-designed TaqMan Low Density Array (TLDA), see supplemental Table 1 for listing of genes. RNA processing and RT-PCR analysis of custom TLDA were performed as previously described^45^. The gene expression panel was normalized using geNorm approach, which identified Gapdh, Ppia and Ppib (from the 9 endogenous control genes included in the custom TLDA)to be used for normalization, as previously described in^45^.

#### The light and dark box test (LDBT) and open field test (OFT)

The LDBT apparatus consisted of one lit compartment (45 × 32 × 32 cm, 50 lux; light box) and one dark compartment (30 × 32 × 32 cm, 5 lux), which were adjacent to each other. A small opening (10 × 15 cm) enabled the rat to transition between the boxes. On the test day, rats were placed in the light compartment and the time spent in each compartment during the 5 min test.

The OFT covered an area of 90 cm × 90 cm, with 40 cm high walls which was divided into a 6 × 6 grid of equally-sized squares using black tape (36 total squares) with 4 squares forming the center; 12 squares forming the middle perimeter; and 20 squares forming the outer perimeter. On the test day, rats were placed in the center and the time spent in the different zones during the 5 min test. Both tests were video-recorded and scored using Anymaze video tracking software (Stoelting, Woods Dale, IL).

#### Elevated plus maze

The elevated plus maze (Hamilton-Kinder San Diego, CA) is 111.76 cm wide x 111.76 cm deep x 85.1 cm tall and each arm is 10.79 cm wide and 50.16 cm long, intersection is 10.79 cm by 10.79 cm, closed walls are 40.00 cm high. On the test day, the animals were placed in the center area of the maze and allowed to explore the open and closed arms for 5 min. The total amount of time spent in each arm was recorded directly to the computer from the infrared beam breaks.

#### Gas infusion procedure for in vivo respiration activity measures

Rats were placed in a clear Plexiglass® cylindrical plethysmograph chamber (i.d. 95 mm, length 260 mm, volume 1.84 l, wall thickness 3 mm) with atmospheric air infused at a flow rate of 2.8 l/min using a Vetamac flowmeter (Rossville, IN) until a steady baseline respiration rate was noted. A differential pressure amplifier (model 24PC01SMT, Honeywell Sensor, Golden Valley, MN) was connected 20 cm away from the start of the output line to a Powerlab data 8/35 acquisition system (ADInstruments, Colorado Springs, CO) for continuous monitoring of respiration. The second T arm connector was opened to the room air. All rats had infusions of the following: (1) 5 min infusion of atmospheric gas (<1% CO_2_, 21% O_2_, 79% N_2_) for baseline measurements, then (2) either the control gas or experimental normoxic, hypercarbic gas (7.5% CO_2_, 21% O_2_, 59% N_2_) for 5 min, and finally, (3) 5 min infusion of atmospheric gas. Respiration rate was assessed by calculating the rate of pressure fluctuations inside the chamber using LabChart software (ADInstruments), and sighing was also visualized using Labchart software. A sigh was defined as an initial respiration that immediately precedes an additional second deeper inspiration. The sigh is followed by an expiration that is larger than usual, and is often accompanied by a post sigh apnea^46^. supplemental Fig. 1 illustrates the changes in the concentrations of CO_2_ and O_2_ that occur prior to, during and at offset of gas infusion.

#### Gas infusion procedure for in vitro measurement of ambient CO_2_ and O_2_ concentrations

Here we used state of the art sensors (www.CO2-meter.com, Osmond Beach, Fl) to measure changes in the concentrations of CO_2_ (Cozir, GSS IR LED sensor, 0-100% range with 3%+/− error) and O_2_ (UV light flux sensor, 0–25% range with 2%+/− error) within a clear custom built Plexiglass® cylindrical plethysmograph chamber (i.d. 95 mm, length 260 mm, volume 1.84 l, wall thickness 3 mm) with atmospheric air infused for 5 min; then 5 min of normoxic 7.5CO_2_; followed by 5 min of atmospheric air. The flow rate was 2.8 l/min using a Vetamac flowmeter (Rossville, IN). The battery operated sensors within the cages were interfaced with Gaslab data acquisition software (www.CO2-meter.com) on a Windows PC.

Initial in vitro assessments of CO_2_ and O_2_ concentration changes following a 5 min infusion of normoxic 7.5% CO_2_ we determined that CO_2_ concentrations went from undetectable levels to 5% within 2 min and 7–7.5% 3–6 min and returned to baseline 4 min following the CO_2_ infusion (F_(11,55)_ = 81.0, *p* < 0.001, Suppl. Fig. 1, *n* = 6). Percent O_2_ concentrations went from 20.6%O_2_ to 20.9%O_2_ during the CO_2_ challenge, then back to 20.6%O_2_ following the CO_2_ gas challenge (F_(11,55)_ = 86.0, *p* < 0.001, Suppl. Fig. 1, *n* = 6).

Supplemental Fig. 1—Line graphs in, respectively, represent the mean ambient concentrations of CO_2_ and O_2_ at 2 min baseline when atmospheric air was infused; 5 min post normoxic 7.5%CO_2_ gas infusion; and 5 min of atmospheric air infusion. # symbol represents within subjects over time significance with a 2 tailed Dunnett’s posthoc test protected by a significant one way ANOVA with each gas treatment as the main factor. Error bars represent the SEM.

Supplemental Fig. 2–a-b represents representative sighs and sigh frequency between genotypes, respectively. (c) are representative respiratory responses to atmospheric air in controls and to 7.5%CO_2_ in each genotype group.

Supplemental Fig. 3–a representative evoked action potential recordings from BLA neurons of WT, SERT^+/−^ and SERT^−/−^ rats; (c) representative spontaneous activity recordings in voltage-clamp mode from BLA neurons of WT, SERT^+/−^ and SERT^−/−^ rats, (c) bar graphs summarizing amplitude and frequency of sEPSCs of WT, SERT^+/−^ and SERT^−/−^ rats. Error bars represent the SEM.

### Shock sensitivity protocol

In order to confirm there were no differences in shock sensitivity between genotypes, rats from each genotype group were placed in the same fear conditioning chambers as described previously. The rats were then exposed to a series of 500 ms shocks starting at 0.1 mA and increasing by 0.1 mA after each 20 s interval. The behavior was recorded for the duration of the experiments and the latency to induce a startle response was then used to determine shock sensitivity.

In this experiment the mean shock needed to induce a startle response was ~0.4 mA with no difference noted between genotypes [Suppl. Fig. 4, WT, *n* = 6; SERT^+/−^ *n* = 5; SERT^−/−^ *n* = 6; F_(2,14)_ = 0.04, *p* = 0.96]. This was also below the 0.8 mA shock used for fear conditioning experiment.

Supplemental Fig. 4*—*Bar graph represents the mean intensity of shock needed to induce a startle response between wildtype (*n* = 6), SERT^+/−^ (*n* = 5) and SERT^−/−^ (*n* = 5) rats. The arrow indicates the shock intensity used for fear conditioning experiment in Fig. 3. Error bars represent the SEM.

Supplementary Table 1*—*List of the 21 serotonin related genes assessed in the BLA using a custom TaqMan RT-PCR array microfluidic card.

## Supplementary information

Supplemental Figure 1

Supplemental Figure 2

Supplemental Figure 3

Supplemental Figure 4

Supplemental Table 1
