# Supplementary figures and images for: Assessment of fear and anxiety associated behaviors, physiology and neural circuits in rats with reduced serotonin transporter (SERT) levels
Source: Transl Psychiatry. 2019 Jan 22;9:33. doi: 10.1038/s41398-019-0368-y (PMC6343029; doi:10.1038/s41398-019-0368-y)

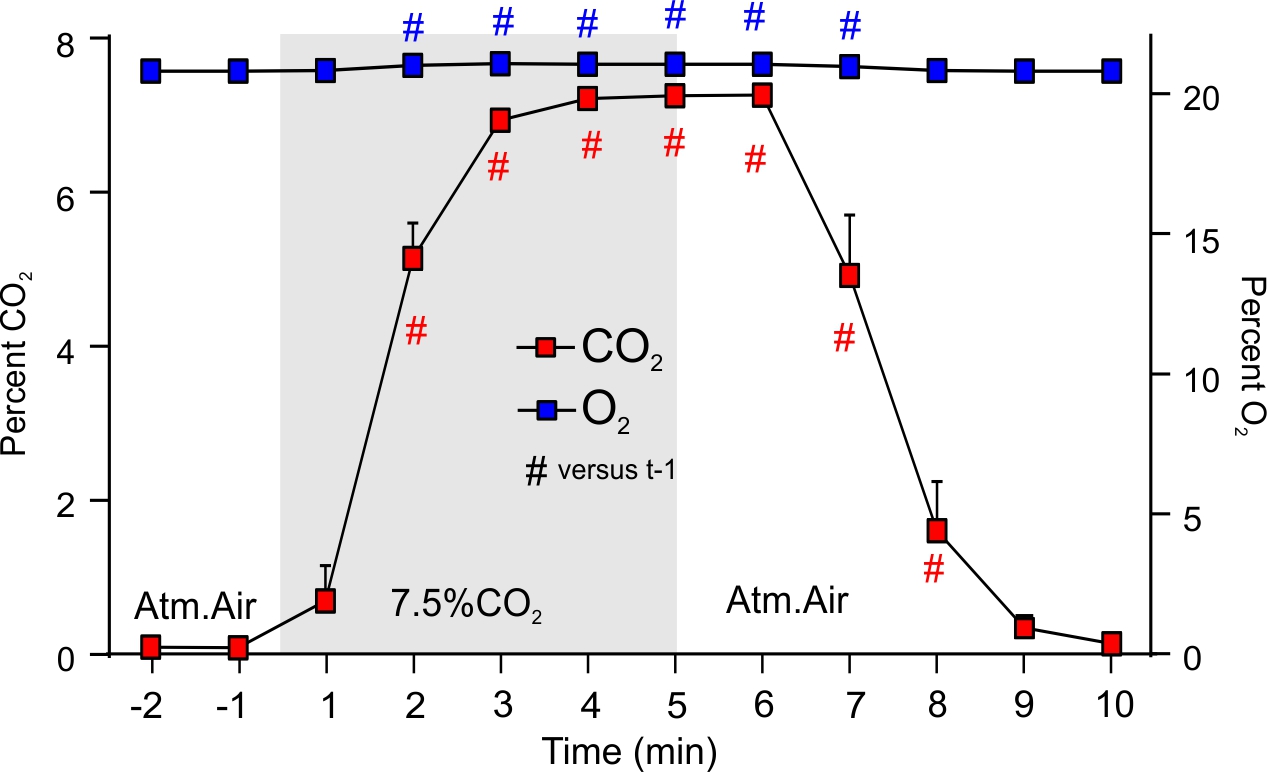

Supplement: Supplementary file 1 — Supplemental Figure 1 [file 41398_2019_368_MOESM1_ESM.jpg]

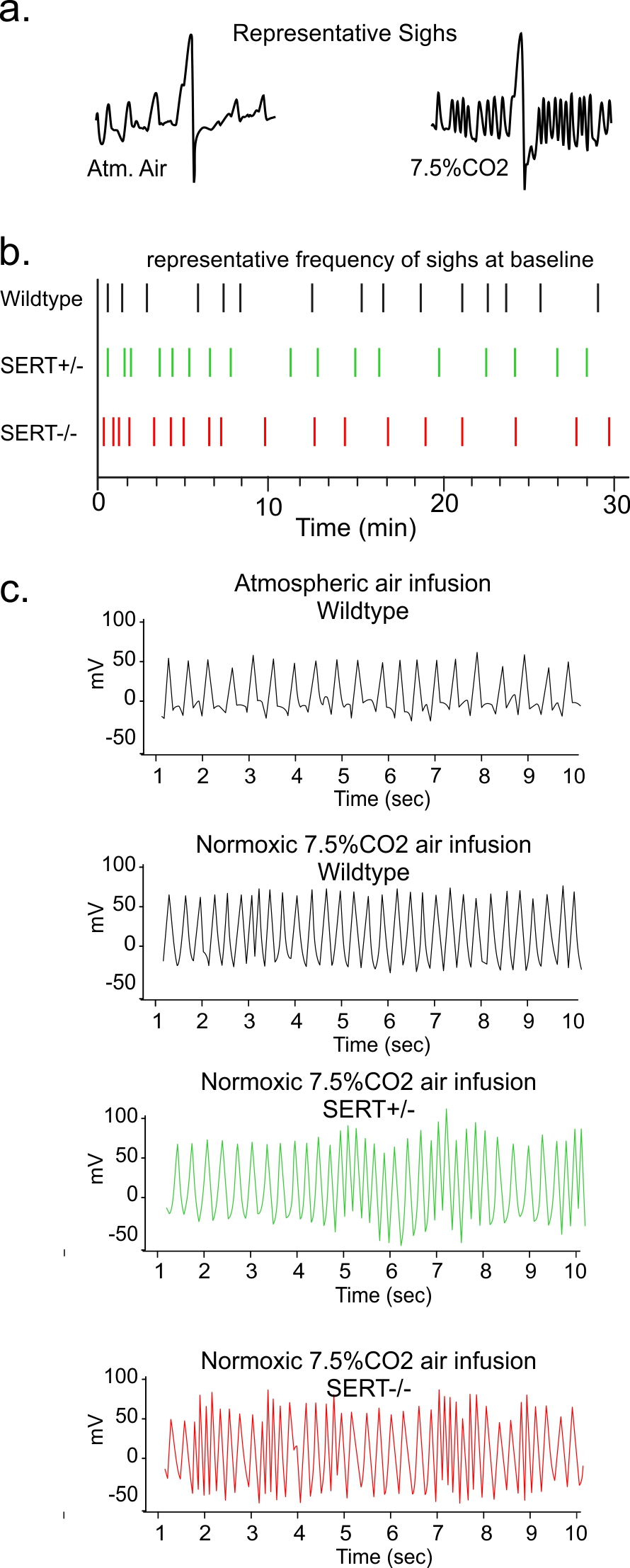

Supplement: Supplementary file 2 — Supplemental Figure 2 [file 41398_2019_368_MOESM2_ESM.jpg]

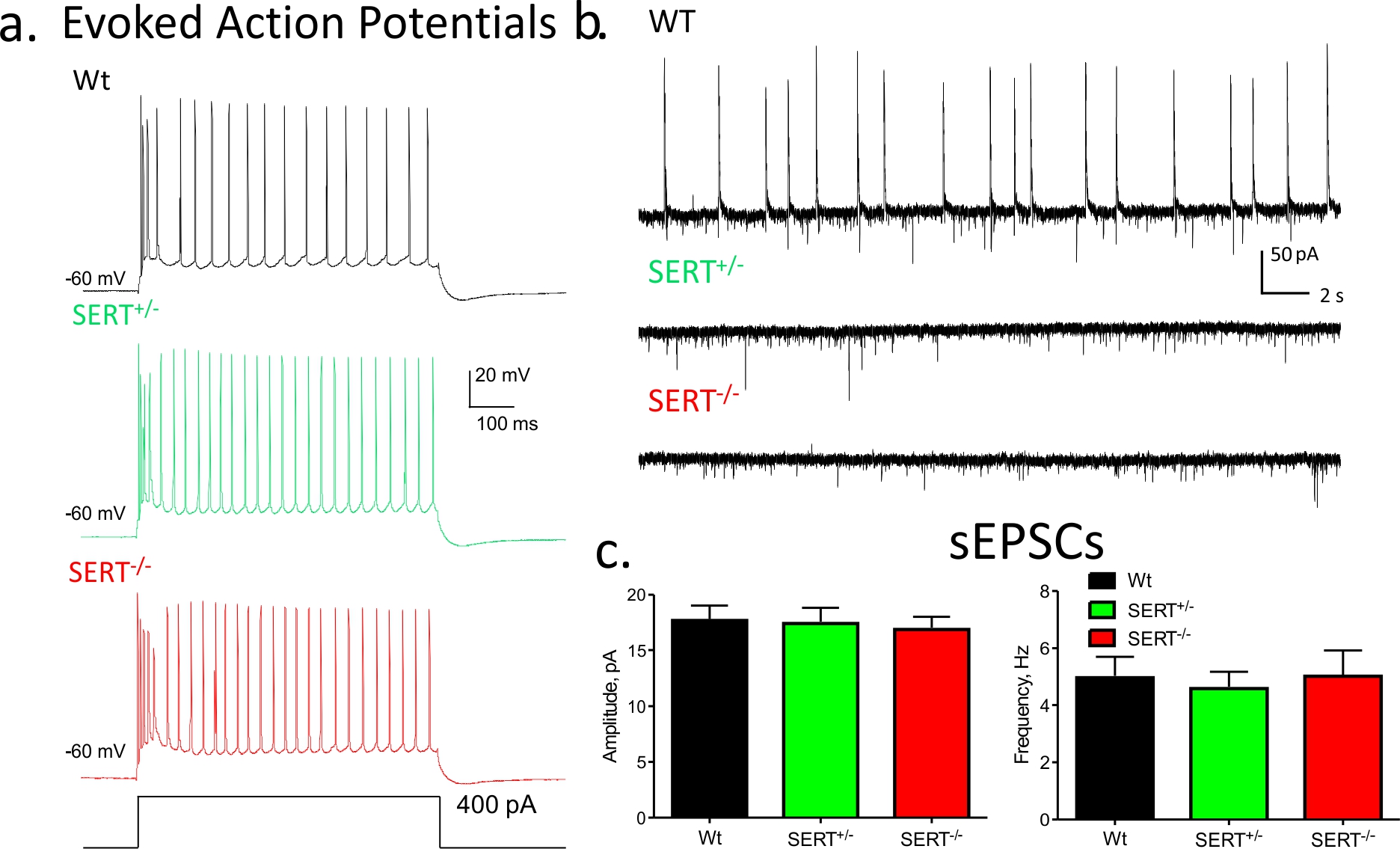

Supplement: Supplementary file 3 — Supplemental Figure 3 [file 41398_2019_368_MOESM3_ESM.jpg]

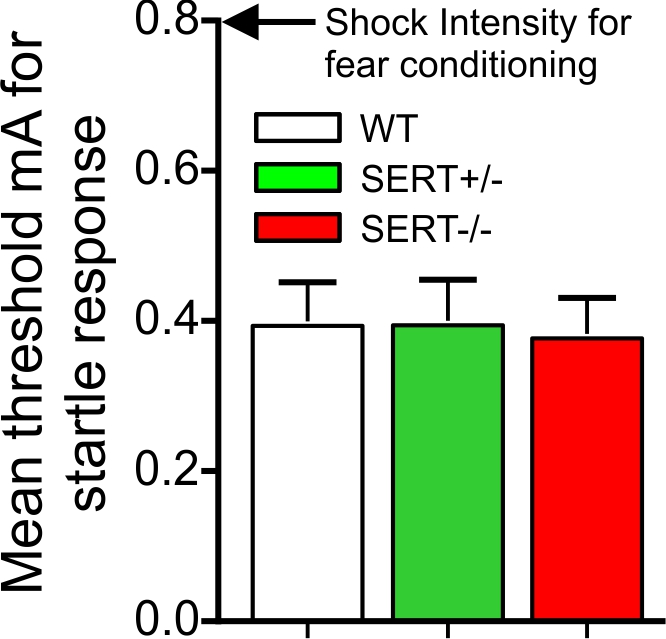

Supplement: Supplementary file 4 — Supplemental Figure 4 [file 41398_2019_368_MOESM4_ESM.jpg]

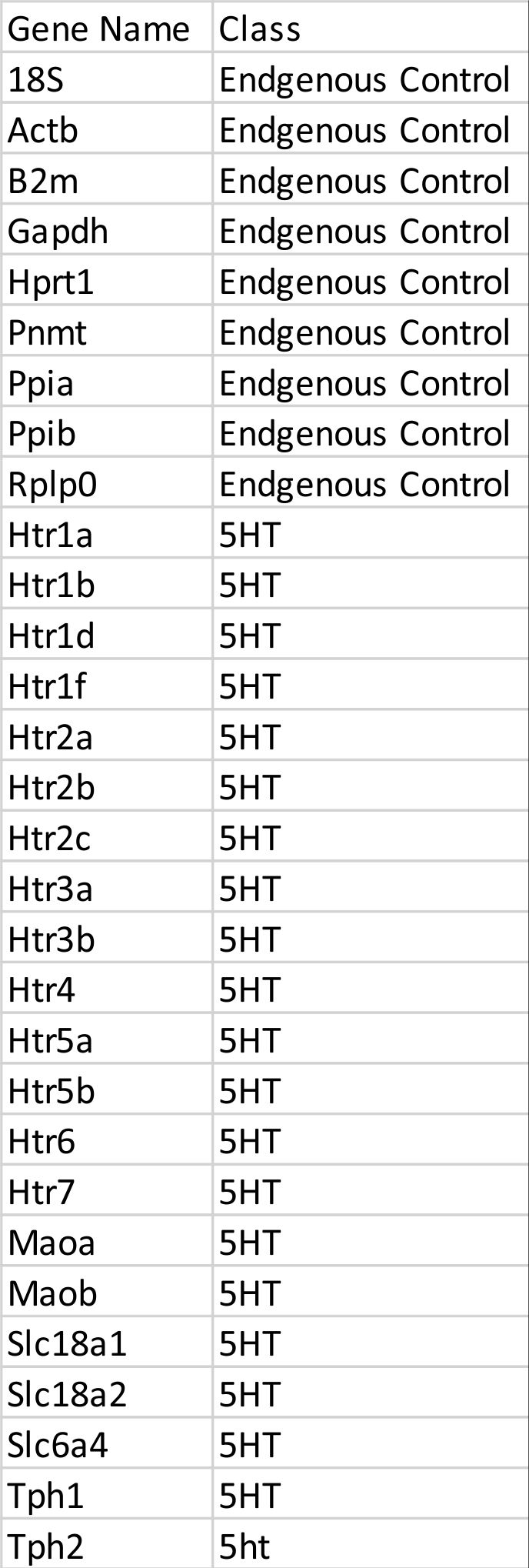

Supplement: Supplementary file 5 — Supplemental Table 1 [file 41398_2019_368_MOESM5_ESM.jpg]
